# Editorial: ESKAPE biofilm: challenges and solutions

**DOI:** 10.3389/fcimb.2023.1253439

**Published:** 2023-08-01

**Authors:** Vishvanath Tiwari

**Affiliations:** Department of Biochemistry, Central University of Rajasthan, Ajmer, Rajasthan, India

**Keywords:** biofilm, DNase, non-thermal plasma, nanotherapeutics, combinatorial therapies

ESKAPE pathogens have gained notoriety due to their ability to evade the effect of antibiotics which has made them a serious public health concern. These bacteria can create a protective barrier i.e. biofilm, that shields them from the immune system and renders an antibiotic ineffective. Biofilms can be defined as complex microbial communities encased in a self-produced matrix and pose significant challenges in healthcare industries, particularly in the context of antibiotic resistance. Among numerous biofilm-forming bacteria, specifically, the ESKAPE pathogens (*Enterococcus faecium, Staphylococcus aureus, Klebsiella pneumoniae, Acinetobacter baumannii, Pseudomonas aeruginosa*, and *Enterobacter sps.*) have emerged as a major threat as they lead to persistent infections and increased mortality rates (Founou et al., 2017). There are different antibiofilm therapies have been investigated (Roy et al., 2018). In this editorial, we delve into the challenges posed by ESKAPE biofilms and explore potential solutions to combat this growing concern.

Biofilms exhibit intricate structures and diverse microbial populations. This heterogeneity contributes to the complexity of eradicating biofilms. Rapid and accurate diagnostics tools to identify biofilms are limited, and therefore, biofilms-related infections often go undetected or misdiagnosed, leading to delayed or inappropriate treatment (Harro et al., 2020). The dearth of effective antimicrobial agents specifically designed to target biofilms is a significant challenge. Novel therapeutic strategies are thus needed to combat ESKAPE biofilms effectively. Alternative therapies, such as the use of antibiotics in combination with adjuvants, antimicrobial peptides, nanoparticles, bacteriophages, and photodynamic light therapies etc., are widely reported (Kaur, 2016; Roy et al., 2018).

Since modern medicines are facing the challenges of antibacterial resistance, and the effectiveness of the currently available antibiotics is declining, causing the rapid emergence of resistant bacteria. Researchers have come up with novel strategies for combating these resistant biofilms by focusing on quorum sensing (QS), and interfering QS mechanisms could give rise to novel compounds to prevent bacterial infections. Recently, quorum sensing inhibitors (QSIs) have been chosen as one of the alternatives to these antimicrobial agents. They can act as the natural immune enhancer and combat disease resistance without any selective pressure among pathogenic bacteria. Different novel targets like electrochemical signalling have been identified in *Acinetobacter baumannii* that may be worked as target for anti-biofilm molecules (Tiwari et al., 2023).

In an interactive review, Venkateswaran et al. discussed the holistic approach that includes the mechanism of infection as well as the recent advancement in preventing and treating infections against ESKAPE pathogens. Their study discussed the quorum sensing circuit of the ESKAPE pathogen that involves the LuxS system in altering antibiotic susceptibility and forming biofilms. Multiple quorum-sensing networks are often involved in these organisms’ biofilm formation process. These quorum-sensing networks can be targeted. Gerdt et al. showed that the inadequacy of quorum-sensing signals by QSI-sensitive bacteria and their cheating mechanisms against the rare QSI-resistant bacteria would inherently reduce the spread of resistance against QSIs targeting QS receptor function (Gerdt and Blackwell, 2014).

Furthermore, in another study conducted by Limayem et al. where the bactericidal effects of silver hydrosol nanotherapeutics against *Enterococcus faecium* drug-resistant biofilms have also been documented. The quantitative concentration response showed that Ag-hydrosol nanoparticles exhibit relatively high antibiofilm activity and low cytotoxicity. Thus, the bioactive Ag-hydrosol NPs can be a promising nanotherapeutic agent against drug-resistant pathogens. In a similar study conducted by Tiwari et al., polyvinylpyrrolidone-capped silver nanoparticles inhibit infection of the carbapenem-resistant strain of *A. baumannii* in the human pulmonary epithelial cells. The study concluded that PVP-AgNPs could be developed as a substitute for carbapenem to control the infection caused by carbapenem-resistant *A. baumannii* (Tiwari et al., 2017).

The trend of therapeutics has shifted towards innovative strategies. Recently, Kašparová et al. showed that non-thermal plasma is effective against resistant biofilms of *Pseudomonas aeruginosa* that inhibit the production of Las-B elastase, protease and pyocyanin which in turn releases biofilm cells. Similarly, in another study, Deng et al. have shown that DNase I significantly inhibit early biofilm formation in *P. aeruginosa* and *Staphylococcus aureus* induced empyema models in a dose-dependent manner.

These strategies, like DNase treatments, non-thermal plasma and silver hydrosol, appear to be a key step in overcoming the drug resistance in ESKAPE pathogens. However, while designing any compound to combat biofilm, observing its proper delivery in any *in-vivo* system at a particular site is always significant. The dosage of the anti-biofilm compound is another crucial factor that needs thorough investigation. In the end, I would also like to thank all the reviewers for their comments that improved our manuscripts and authors for their contributions. We hope that this Research Topic will inspire scientists from different fields of research focused on biofilm.

## Author contributions

VT: Data curation, Formal Analysis, Funding acquisition, Investigation, Resources, Validation, Writing – original draft, Writing – review & editing.
